# Robot-Assisted Pedicle Screw Placement Led to Lower Screw Loosening Rate than Fluoroscopy-Guided Technique in Transforaminal Lumbar Interbody Fusion for Lumbar Degenerative Disease: A Single-Center Retrospective Study

**DOI:** 10.3390/jcm11174989

**Published:** 2022-08-25

**Authors:** Yen-Po Lai, Yu-Hsien Lin, Yun-Che Wu, Cheng-Min Shih, Kun-Hui Chen, Cheng-Hung Lee, Chien-Chou Pan

**Affiliations:** 1 Department of Orthopedics, Taichung Veterans General Hospital, Taichung 40705, Taiwan; 2 Department of Physical Therapy, Hungkuang University, Taichung 43304, Taiwan; 3 College of Medicine, National Chung Hsing University, Taichung 40227, Taiwan; 4 Department of Computer Science & Information Engineering, College of Computing and Informatics, Providence University, Taichung 43301, Taiwan; 5 Department of Biomedical Engineering, College of Intelligent Technology, Hungkuang University, Taichung 43304, Taiwan; 6 Department of Food Science and Technology, Hungkuang University, Taichung 43304, Taiwan; 7 Department of Rehabilitation Science, Jenteh Junior College of Medicine, Nursing and Management, Miaoli 35664, Taiwan

**Keywords:** robot-assisted pedicle screw placement, screw loosening rate, transforaminal lumbar interbody fusion, lumbar degenerative disease

## Abstract

Robot-assisted pedicle screw placement for spine surgery has become popular in recent years. This study compares clinical, radiographic outcomes and the screw loosening rate between robot-assisted and fluoroscopy-guided pedicle screw placement in patients who underwent transforaminal lumbar interbody fusion (TLIF). We retrospectively examined 108 patients with the degenerative lumbar disease who underwent TLIF. According to whether the robotic system was used, patients were assigned to either the robot-assisted (Ro TLIF, *n* = 29) or fluoroscopy-guided TLIF (FG TLIF, *n* = 79) group. Radiographic parameters and patient-reported outcomes, including leg and back pain visual analog scale (VAS) and Oswestry Disability Index (ODI), were assessed. Loosening signs were noted in 48 out of 552 pedicle screws. The screw loosening rate was higher in the FG TLIF (10.2%) than Ro TLIF group (4.3%). A significant correlation was found between screw loosening and age, the number of level(s) fused, and the ratio of the average distance from the pedicle screw to the upper endplate to vertebral body height. VAS-leg, VAS-back, and ODI showed significant improvements in both groups postoperatively (all *p* < 0.05). These results indicated that robot-assisted pedicle screw placement in TLIF had a lower screw loosening rate and similar patient-reported outcomes compared with the fluoroscopy-guided technique.

## 1. Introduction

Pedicle screw placement for spinal fusion surgery has been widely performed since its introduction by Boucher in the 1950s [1]. In recent years, robotic systems have been developed to assist pedicle screw placement in spinal surgery. According to previous literature, robot-assisted pedicle screw placement has greater screw accuracy [2,3,4,5], fewer proximal facet joint violations [2,6], reduced radiation exposure [2,5,7,8], and shorter length of hospital stay [7]. However, only a few studies have compared the postoperative clinical and radiographic outcomes between robot-assisted pedicle screw placement and fluoroscopy-guided technique. We inferred that, with the aid of a robotic system, we could design pedicle screws closer to the upper endplate and the anterior body, which is also known as the cortical bone. Theoretically, pedicle screws provide a greater fixing force on the cortical bone, thus lowering the screw loosening rate. Pedicle screw loosening is one of the most frequently reported complications in patients who have undergone transforaminal lumbar interbody fusion (TLIF). Several factors may contribute to screw loosening, including diabetes mellitus, a higher number of fused levels, decreased bone radiodensity in Hounsfield units, posterior fusion performed without anterior support, unrestored alignment in the case of spondylolisthesis, and lumbosacral fusion [9,10,11]. Patients with pedicle screw loosening are also reported to have worse clinical results including Oswestry Disability Index (ODI) and pain visual analog scale (VAS) [12]. This study aimed to compare the postoperative clinical and radiographic outcomes of patients who underwent TLIF using either robot-assisted or fluoroscopy-guided pedicle screw placement. We hypothesized that robotic assistance would result in a lower screw loosening rate compared with fluoroscopic guidance during pedicle screw placement.

## 2. Materials and Methods

### 2.1. Study Design and Patients

This was a retrospective single-center cohort study. This study was approved by the institutional review board of our institution (approval no.: CE21337A). We identified all patients with the lumbar degenerative disease who underwent TLIF at our institution between May 2018 and September 2020. The operations were performed by three spinal surgeons with more than 20 years of experience as attending physicians. The inclusion criteria were as follows: (1) patients with the degenerative lumbar disease who underwent TLIF at our hospital, (2) 18 years old or older, and (3) regular follow-up at our outpatient department for at least 12 months. The exclusion criteria were as follows: (1) age < 18 years, (2) follow-up period < 12 months or loss to follow-up, and (3) underwent TLIF due to infection, revision, tumor, or trauma.

The patients were assigned to the robot-assisted TLIF (Ro TLIF) group or the fluoroscopy-guided TLIF (FG TLIF) group, according to the method used for pedicle screw placement.

Baseline data included age, sex, body mass index (BMI), diabetes mellitus (DM), diagnosis, number of level(s) fused, cage type (polyetheretherketone (PEEK) or trabecular metal (TM) cage), screw type (solid or hollow screw), surgical segment(s), estimated blood loss, surgical time, and length of hospital stay.

### 2.2. FG TLIF Procedure

The general steps of the FG TLIF procedure are as follows: A posterior midline incision was made. Paravertebral muscles were dissected from the spinous process, lamina, facet, and transverse process to expose the anatomical landmarks for pedicle screw entry points. Screw diameter was determined by pedicle size on preoperative MRI. An awl was used for entry point (located at cranial third of the junction of transverse process and facet joint) followed by probing the pedicle tract to the vertebral body. Screw length was determined by measuring the probe length in the bone tunnel. Guide pins for pedicle screws were placed through the tract, and their positions were confirmed by fluoroscopy. Decompression was performed using laminotomy, laminectomy, foraminotomy, facetectomy, discectomy, or a combination of the above procedures according to clinical circumstances. Endplates were prepared, followed by the placement of an interbody device with bone grafts. Pedicle screws were inserted via guide pins with hollow screws or under direct vision with solid screws, and the rods were connected.

### 2.3. Ro TLIF Procedure

**Figure 1:**
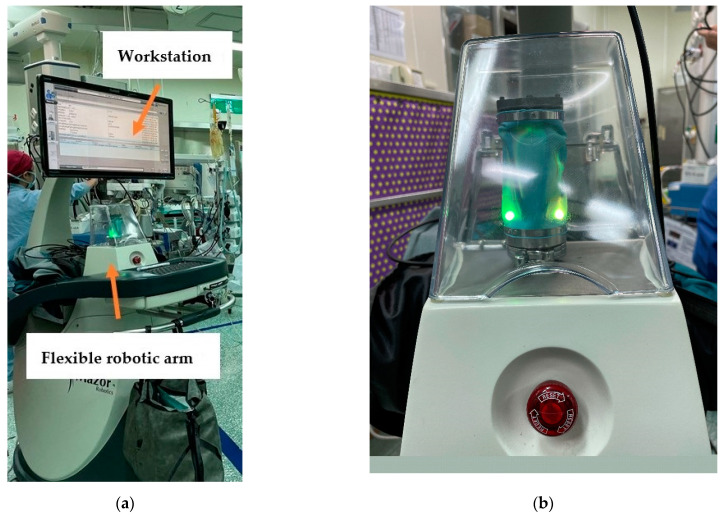

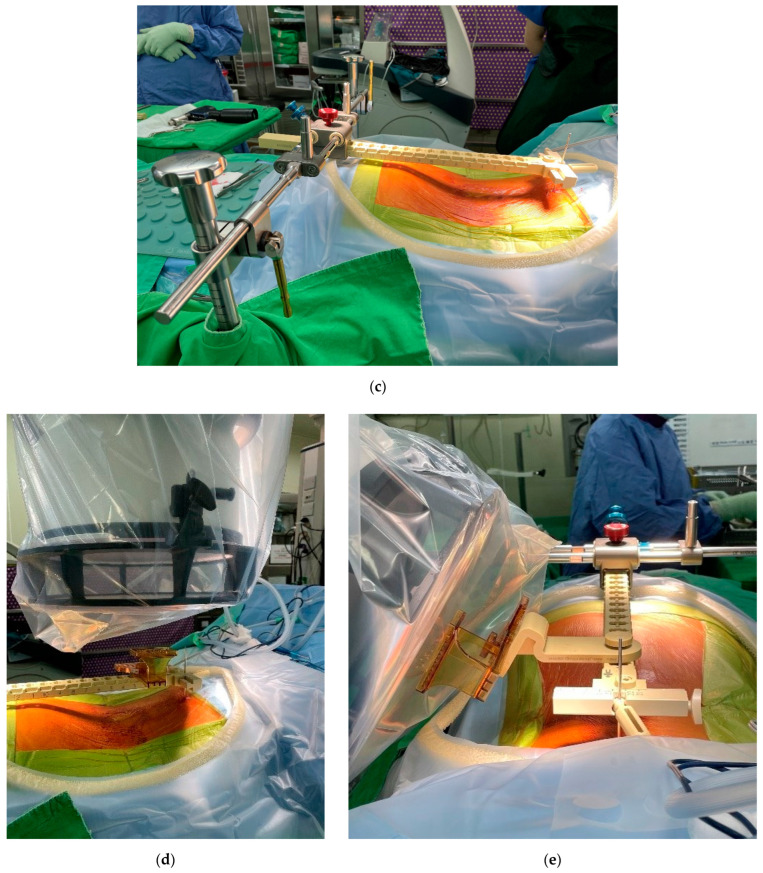

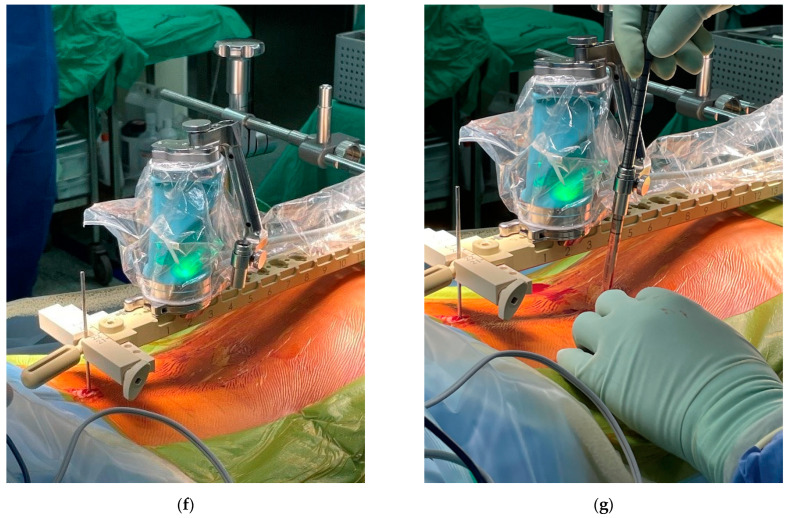
Mazor Robotics Renaissance^®^ system (Medtronic, Denver, CO, USA). (**a**) Workstation and flexible robotic arm. (**b**) Flexible robotic arm. (**c**) A multi-directional bridge connecting to the surgical table and a spinous process headpin. (**d**) Anteroposterior fluoroscopic image. (**e**) 60° oblique fluoroscopic image. (**f**) Flexible robotic arm running to the planned position. (**g**) Cannula placed on the robotic arm.

The Mazor Robotics Renaissance^®^ system (Medtronic, Denver, CO, USA) was used for the Ro TLIF procedure. The system is composed of a flexible robotic arm and a workstation (Figure 1a,b). All patients underwent preoperative spine CT at the index level of surgery, and the images were imported to the workstation. The pedicle screw length, diameter, trajectory, and rod length were planned preoperatively. We intended to design the pedicle screws closer to upper endplate. Patients were placed in the prone position under general anesthesia during the operation. A posterior midline incision was then made. The exposure of anatomical landmarks to the pedicle screw entry points was completed. Several methods can be used to attach the robotic arm to the patient, including bilateral posterior superior iliac spine Schanz screws plus a spinous process headpin with a link to the Hover-T bridge, a spinous process clamp, or a multi-directional bridge (Figure 1c) connecting to the surgical table and a spinous process headpin. The 3D marker is fixed for registration. Anteroposterior and 60° oblique fluoroscopic images (Figure 1d,e) were taken and imported to the robotic workstation to finish the registration process. The two aforementioned images were matched with the preoperative CT scan, and the surgeon confirmed the correct registration for each vertebra at the workstation.

**Figure 2:**
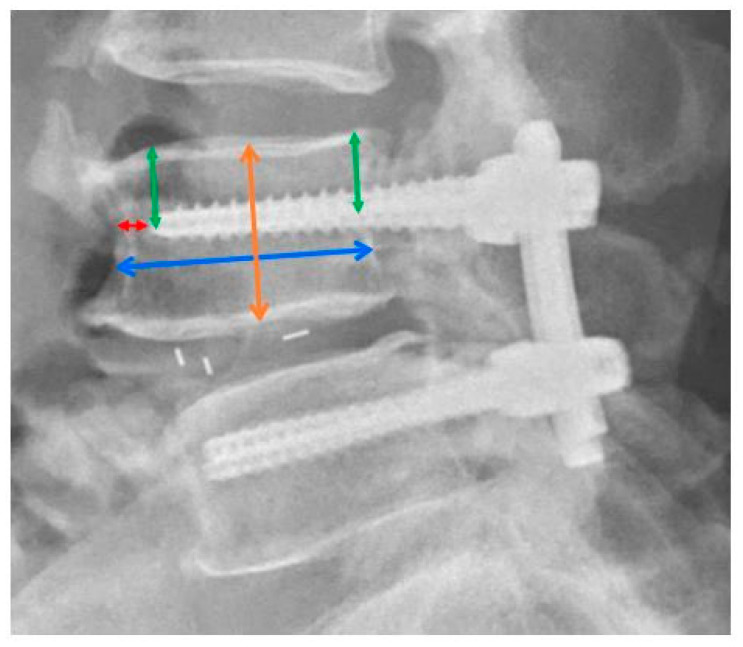
Parameter measurements of pedicle screws. Green arrow: Screw tip to upper endplate and screw to upper endplate at the posterior body level; Red arrow: Screw tip to body anterior cortex; Orange arrow: Vertebral body height; Blue arrow: Vertebral body anteroposterior diameter.

The steps for pedicle screw guide pin insertion using the Mazor Robotics Renaissance^®^ system were as follows: (1) the flexible robotic arm was placed in the planned position (Figure 1f); (2) the cannula and drill guide were placed on the robotic arm (Figure 1g); then advanced to the entry point; (3) drilling was performed at the entry point with a 3 mm diameter drill to 3 cm in depth; and (4) placement of guide pins was performed. After inserting all the guide pins, their positions were checked using an image intensifier. Next, the robotic arm and bridge were removed. Discectomy was done and endplates were prepared, followed by the placement of an interbody device by free hand with bone grafts. Decompression was completed, followed by placing pedicle screws through guide pins and connecting the rods.

### 2.4. Measurements of Radiographic Parameters

L-spine anteroposterior and lateral plain radiographs were obtained on the first postoperative day, as per our standard protocol. We measured several parameters of each pedicle screw on the L-spine lateral view using the Picture Archiving and Communication System Ultraquery (Taiwan Electronic Data Processing, New Taipei City, Taiwan), including the distance from the pedicle screw tip to the upper endplate, pedicle screw to the upper endplate at the posterior body level, and pedicle screw tip to the vertebral body anterior cortex, vertebral body height, and vertebral body anteroposterior diameter (Figure 2). All parameters were measured independently by two orthopedic surgeons. The data was collected and calculated for interclass correlation, and the intraclass correlation coefficient (ICC) was used to assess the reliability of the measurement. We calculated the average distance from the pedicle screw to the upper endplate and the ratio of (1) distance from the pedicle screw to the upper endplate/vertebral body height and (2) distance from the pedicle screw tip to the vertebral body anterior cortex/vertebral body anteroposterior diameter.

### 2.5. Definition of Screw Loosening and Cage Subsidence

**Figure 3:**
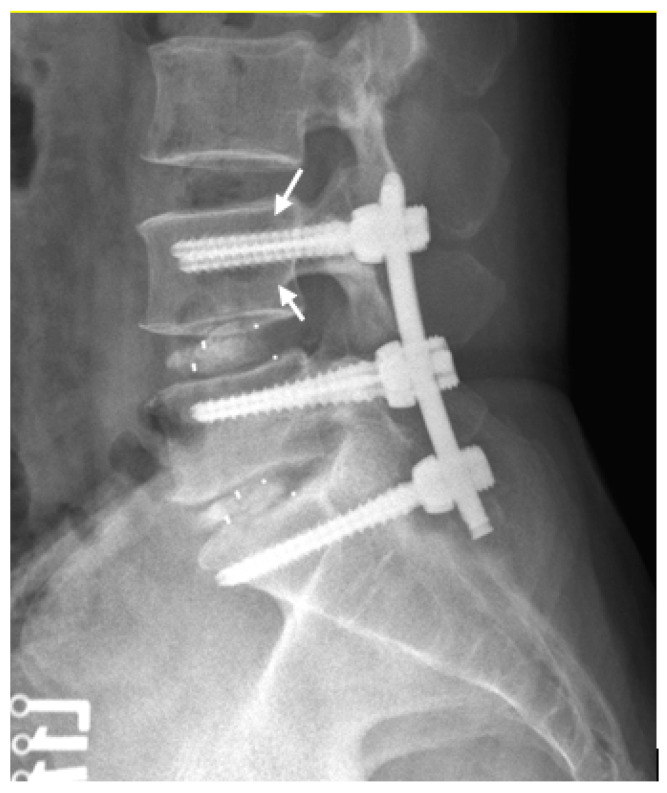
Lumbar spine lateral view after L4–S1 TLIF surgery of a 53-year-old man. Six months postoperatively, the arrows revealed radiolucency ≥1 mm around pedicle screws of L4, indicating screw loosening.

Screw loosening is defined as a minimal thickness of 1 mm radiolucent zone around the edges of pedicle screws on plain radiography [9,13,14,15,16,17] (Figure 3). Cage subsidence is defined as the subsidence of the superior and inferior part of the cage into the vertebral body on standing neutral lateral plain radiographs [18]. Any occurrence of screw loosening or cage subsidence was noted on plain radiographs obtained at the last follow-up (postoperative 12 months).

### 2.6. Clinical Outcomes

Patient-reported outcomes, including the back and leg pain visual analog scale (VAS) and the Oswestry Disability Index (ODI), were recorded on the day before surgery and at 1, 3, 6, and 12 months postoperatively.

Further, complications, including urinary tract infection, surgical site infection, screw loosening, cage subsidence, and broken rod or screw, were noted.

### 2.7. Statistical Analysis

Categorical variables between the groups were compared using the chi-square test. Continuous variables were compared using the Mann–Whitney U test. Differences in the patient-reported outcomes in the same group were calculated using the Friedman test. The association between screw loosening rate and potential risk factors was assessed using logistic regression analysis. A correlation analysis was conducted using the Pearson correlation coefficient to validate the intraobserver and interobserver reliability. Statistical significance was set at *p* < 0.05. All statistical analyses were performed using IBM SPSS Statistics, version 22.0 (IBM Corp., Armonk, NY, USA).

## 3. Results

### 3.1. Patients’ Profile

A total of 185 consecutive patients underwent TLIF at our facility between May 2018 and September 2020. Twenty-nine cases of revision and one case of infection were excluded. A total of 155 patients met our inclusion criteria and were assigned to the Ro TLIF or the FG TLIF group according to whether the robotic system was used for pedicle screw placement. Seven patients in the Ro TLIF group and 24 in the FG TLIF group had a follow-up period of fewer than 12 months and were excluded. Two patients in the Ro TLIF group and 14 in the FG TLIF group performed by two spinal surgeons less than 10 years of experience as attending physicians were also excluded. Finally, 29 patients were assigned to the Ro TLIF group, and 79 patients were assigned to the FG TLIF group.

Cohort characteristics are presented in Table 1. The majority of the patients in each group were female and were in their 70s. Most of the patients in each group underwent single-level TLIF. There were two differences between the two groups: spondylolisthesis was the most prevalent diagnosis in the FG TLIF group, whereas spinal stenosis was most prevalent in the Ro TLIF group. As for pedicle screw type, 75.9% of patients in the FG TLIF group were solid screws, while all patients in the Ro TLIF group were hollow screws under the recommended procedure of Mazor Robotics Renaissance^®^ system. There were no significant differences in age, sex, BMI, DM, number of level(s) fused, surgical segment(s), cage type, preoperative pain VAS-leg, preoperative pain VAS-back, or preoperative ODI (Table 1).

### 3.2. Perioperative and Postoperative Outcomes

Perioperative and postoperative outcomes are shown in Table 2. There were no significant differences in the estimated blood loss for single-level TLIF (366.7 mL, 400.0 mL; *p* = 0.677), operative time for single-level TLIF (225.0 min, 259.0 min; *p* = 0.101), and length of hospital stay (6 days, 7 days; *p* = 0.090) between the FG TLIF and Ro TLIF groups. In the FG TLIF group, 14 patients had screw loosening, 2 had cage subsidence, 2 had superficial wound infection, 1 had urinary tract infection, and 1 had a broken rod during the follow-up period. In the Ro TLIF group, three patients had screw loosening, three had cage subsidence, two had superficial wound infection, and two had urinary tract infection. A total of 19 patients (24.1%) in the FG TLIF group and 7 patients (24.1%) in the Ro TLIF group had complications (some patients might have had two or more of the above complications), and no significant difference was found between the two groups (*p* = 1.000). The only complication requiring revision surgery in the patient was a broken rod in the FG TLIF group.

### 3.3. Pedicle Screw Measurements in the FG TLIF and Ro TLIF Groups

There was a total of 412 pedicle screws in the FG TLIF group and 140 screws in the Ro TLIF group. The ratio of the distance from the pedicle screw to upper endplate to the vertebral body height was higher in the FG TLIF group than that in the Ro TLIF group (0.39 and 0.35, *p* < 0.001). The ratio of the distance from the pedicle screw tip to the anterior cortex to the anteroposterior diameter of the vertebral body showed no significant difference between the FG and Ro TLIF groups (0.14, 0.12; *p* = 0.124).

During the follow-up period, 48 of 552 (8.7%) pedicle screws showed signs of loosening on plain radiography (defined as a minimal thickness of 1 mm radiolucency around the edges) [9,13,14,15,16,17]. The screw loosening rate was higher in the FG TLIF than in the Ro TLIF group (42/412, 10.2%; 6/140, 4.3%; *p* = 0.049). The results are presented in Table 3.

Analysis of the screw loosening and non-screw loosening groups showed that the ratio of the distance from pedicle screw to upper endplate to the vertebral body height was higher in the screw loosening group than non-screw loosening group (0.40 and 0.37, *p* < 0.001) (Table 4). The ratio of the distance from the pedicle screw tip to the anterior cortex to the vertebral body anteroposterior diameter showed no significant difference between the screw loosening and non-screw loosening groups (0.16, 0.13; *p* = 0.100).

### 3.4. Intraclass Correlation Coefficient (ICC) for Pedicle Screw Measurements

The intraobserver and interobserver ICCs were excellent (ICC > 0.9) for all measurements.

### 3.5. Screw Loosening Risk Factor Variables

The correlation between potential risk factors and pedicle screw loosening rate was established using a logistic regression model (Table 5). The screw loosening rate showed a positive correlation with age, the number of level(s) fused, and the ratio of the distance from pedicle screw to upper endplate to vertebral body height. Other variables including sex, BMI, diagnosis, screw type, cage type, and the ratio of the distance from screw tip to anterior cortex to vertebral body AP diameter were not risk factors for screw loosening.

### 3.6. Patient-Reported Outcomes

The preoperative ODI (57.78, 57.78; *p* = 0.868) and VAS scores for back pain (8.0, 8.0; *p* = 0.972) and leg pain (8.0, 8.0; *p* = 1.000) were not significantly different between the FG and Ro TLIF groups (Table 1).

**Figure 4:**
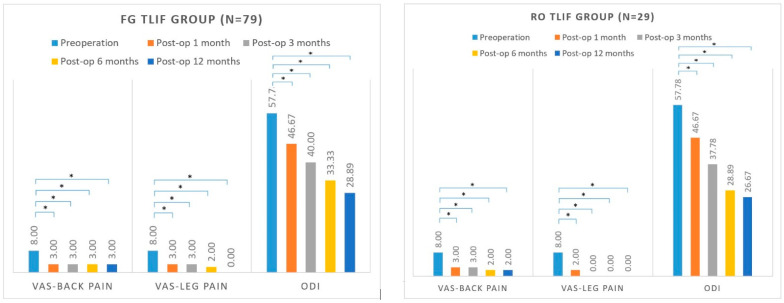
Patient-reported outcomes of FG TLIF and Ro TLIF group. * *p* < 0.05.

The VAS score for back pain in the FG TLIF group decreased from 8.0 preoperatively to 3.0 at 1 month postoperatively, 3.0 at 3-month follow-up, 3.0 at 6-month follow-up, and 3.0 at the 12-month follow-up. The VAS score for leg pain in the FG TLIF group decreased from 8.0 preoperatively to 3.0 at 1 month postoperatively, 3.0 at 3-month follow-up, 2.0 at 6-month follow-up, and 0.0 at 12-month follow-up. The ODI score in the FG TLIF group decreased from 57.78 preoperatively to 46.67 at 1 month postoperatively, 40.00 at 3-month follow-up, 33.33 at 6-month follow-up, and 28.89 at 12-month follow-up (Figure 4).

The VAS score for back pain in the Ro TLIF group decreased from 8.0 preoperatively to 3.0 at 1 month postoperatively, 3.0 at 3-month follow-up, 2.0 at 6-month follow-up, and 2.0 at 12-month follow-up. The VAS score for leg pain in the Ro TLIF group decreased from 8.0 preoperatively to 2.0 at 1 month postoperatively, 0.0 at 3-month follow-up, 0.0 at 6-month follow-up, and 0.0 at 12-month follow-up. The ODI score in the Ro TLIF group decreased from 57.78 preoperatively to 46.67 at 1 month postoperatively, 37.78 at 3-month follow-up, 28.89 at 6-month follow-up, and 26.67 at 12-month follow-up (Figure 4).

VAS for back pain, VAS for leg pain, and ODI showed significant improvements in both groups postoperatively (all *p* < 0.05).

As for grade of improvements, there were no significant differences between FG TLIF and Ro TLIF group at 1 month, 3 months, 6 months, and 12 months postoperatively. Table 6 shows the median, 25th percentile, and 75th percentile of these variables. 

## 4. Discussion

Robot-assisted pedicle screw placement has become popular in recent years, and it has been proven to have greater screw accuracy [2,3,4,5], fewer proximal facet joint violations [2,6], and reduced radiation exposure [2,5,7,8].

This study found that robot-assisted pedicle screw placement in TLIF patients had similar blood loss, operative time, length of hospital stay, and complication rate compared with fluoroscopy-guided pedicle screw placement. However, the screw loosening rate was lower in the robot-assisted pedicle screw placement group (4.3%) than in the fluoroscopy-guided pedicle screw placement group (10.2%). Ohba, T. et al. reported an overall screw loosening rate about 15.2% (44 out of 290 pedicle screws) in patients who underwent minimally invasive lumbar or thoracic spinal stabilization [12]. Other studies revealed pedicle screw loosening rate ranging from 1% to 15% [10,11]. In our study, the overall pedicle screw loosening rate was 8.7% (48/552), which was similar with other studies. In previous literature, screw loosening was associated with many complications, such as non-union, screw breakage, and pseudoarthrosis [10,19]. Risk factors for pedicle screw loosening have been reported to be older patient age, lower bone density [10], higher number of fused levels [11], and radiographic factor like lower axial trajectory angles [12]. In our study, we found that screw loosening was associated with older age, higher number of fused levels, and ratio of the distance from the screw to the upper endplate to the vertebral body height. Older age may be associated with lower bone marrow density. However, dual-energy X-ray absorptiometry (DXA) for diagnosis of osteoporosis was not evaluated in all patients underwent surgery in our study due to the retrospective nature and might cause bias.

To the best of our knowledge, this is the first study to report the relationship between the distance from the pedicle screw to the vertebral body cortex and the screw loosening rate. We found that the ratio of the distance from the screw to the upper endplate to the vertebral body height positively correlated with the screw loosening rate. When using the fluoroscopy-guided technique, the probe tended to “slide” through the soft part of the pedicle, making it difficult to get closer to the upper endplate. Thus, the distance of the pedicle screw to the upper endplate might be larger when the screw is fixed on less dense bone, resulting in a higher loosening rate. With the robotic system, we could preoperatively design a pedicle screw trajectory closer to the upper endplate; thus, we were able to overcome this problem. In a cadaveric study, the novel hollow screw was less resistant to loosening when compared with a conventional solid pedicle screw [20]. In our Ro TLIF group, we used hollow screws for all patients based on standard procedures. However, the Ro TLIF group had lower screw loosening rate. This means that making screw trajectory closing to upper endplate is a very important factor to lower screw loosening rate.

Patient-reported outcomes showed significant postoperative improvement in both Ro TLIF and FG TLIF groups. The complication rates were also similar between the two groups. Only one complication (broken rod) required revision surgery in the FG TLIF group. Kim et al. [21] found that patients with screw loosening had slightly worse clinical outcome scores, though not statistically significant. Maybe a larger study population would demonstrate a significant difference. In our study, patients in the FG TLIF group had a higher screw loosening rate, but the clinical outcomes were similar between the two groups. The relationship between pedicle screw loosening and clinical outcomes requires further investigation in future studies or a larger patient number is required to address the difference.

This study had several limitations. First, this was a retrospective study, and might have a selection bias. Second, only a limited number of patients were included in the Ro TLIF group. Third, robot-assisted pedicle screw placement was a newly introduced technique at our facility, and the learning curve was not evaluated. Finally, patients underwent operations performed by different surgeons, and different implants (pedicle screw and cage type) were used. Although similar procedures and techniques were preformed, experimental confounding effects may have existed.

## 5. Conclusions

The screw loosening rate was lower in the robot-assisted TLIF group than in the fluoroscopy-guided TLIF group; this may be associated with placing pedicle screws closer to the upper endplate. While performing pedicle screw insertion in future operations, designing a screw trajectory closer to upper endplate may be considered. Although no differences were detected between the robot-assisted and fluoroscopy-guided TLIF patient groups at any of the follow-up periods, there was a highly significant (*p* < 0.05) improvement in all patient-reported outcomes.

## Figures and Tables

**Table 1:** Demographic and baseline characteristics of patients.

	FG TLIF Group (*n* = 79)	Ro TLIF Group (*n* = 29)	*p* Value
Age (years)	64.0 [54.5, 71.0]	66.0 [60.0, 76.5]	0.213
Sex			0.700
Female (%)	46 (58.2%)	15 (51.7%)	
Male (%)	33 (41.8%)	14 (48.3%)	
BMI (kg/m^2^)	25.9 [23.4, 29.0]	27.4 [23.3, 30.6]	0.448
DM	21 (26.6%)	7 (24.1%)	0.993
Diagnosis			0.014 *
Spinal stenosis (%)	21 (26.6%)	17 (58.6%)	
Spondylolisthesis (%)	44 (55.7%)	10 (34.5%)	
Spondylolysis (%)	8 (10.1%)	2 (6.9%)	
Other degenerative disease (%)	6 (7.6%)	0 (0.0%)	
Number of level(s) fused			0.499
One (%)	39 (49.4%)	18 (62.1%)	
L1/L2	1 (1.3%)	0 (0.0%)	
L2/L3	2 (2.5%)	0 (0.0%)	
L3/L4	1 (1.3%)	2 (6.9%)	
L4/L5	22 (27.8%)	12 (41.4%)	
L5/L6	0 (0.0%)	1 (3.4%)	
L5/S1	13 (16.5%)	3 (10.3%)	
Two (%)	32 (40.5%)	9 (31.0%)	
L2/L3/L4	1 (1.3%)	1 (3.4%)	
L3/L4/L5	14 (17.7%)	5 (17.2%)	
L4/L5/L6	0 (0.0%)	1 (3.4%)	
L4/L5/S1	17 (21.5%)	2 (6.9%)	
Three (%)	8 (10.1%)	2 (6.9%)	
L2/L3/L4/L5	2 (2.5%)	0 (0.0%)	
L3/L4/L5/S1	6 (7.6%)	1 (3.4%)	
L4/L5/S1/S2	0 (0.0%)	1 (3.4%)	
Cage type			1.000
Trabecular Metal (%)	19 (24.1%)	7 (24.1%)	
Polyetheretherketone (%)	60 (75.9%)	22 (75.9%)	
Screw type			<0.001 *
Solid (%)	60 (75.9%)	0 (0.0%)	
Hollow (%)	19 (24.1%)	29 (100.0%)	
Pre-op pain VAS—leg	8.0 [7.0, 10.0]	8.0 [6.0, 9.5]	1.000
Pre-op pain VAS—back	8.0 [6.0, 9.0]	8.0 [6.0, 9.0]	0.972
Preoperative ODI	57.78 [51.11, 64.44]	57.78 [47.78, 71.11]	0.868

FG, fluoroscopy-guided; Ro, robot-assisted; TLIF, transforaminal lumbar interbody fusion; BMI, body mass index; DM, diabetes mellitus; Pre-op, preoperative; VAS, visual analog scale; ODI, Oswestry Disability Index. * *p* < 0.05.

**Table 2:** Perioperative and postoperative outcomes.

	FG TLIF Group (*n* = 79)	Ro TLIF Group (*n* = 29)	*p* Value
Blood loss for single-level TLIF (mL)	366.7 [300.0, 600.0]	400.0 [250.0, 675.0]	0.677
Operative time for single-level TLIF (min)	225.0 [170.0, 293.0]	259.0 [222.8, 296.5]	0.101
Length of hospital stay (days)	6.0 [5.0, 8.0]	7.0 [6.0, 8.0]	0.090
Complication (%)	19 (24.1%)	7 (24.1%)	1.000
Complication requiring revision surgery (%)	1 (1.3%)	0 (0%)	1.000

FG, fluoroscopy-guided; Ro, robot-assisted; TLIF, transforaminal lumbar interbody fusion.

**Table 3:** Parameters of pedicle screws in FG TLIF and Ro TLIF group.

	FG TLIF Group (*n* = 412)	Ro TLIF Group (*n* = 140)	*p* Value
Distance from pedicle screw to upper endplate/vertebral body height	0.39 [0.36, 0.42]	0.35 [0.29, 0.37]	<0.001 *
Distance from screw tip to anterior cortex/vertebral body AP diameter	0.14 [0.06, 0.22]	0.12 [0.04, 0.19]	0.124
Screw loosening (%)	42 (10.2%)	6 (4.3%)	0.049 *

Values are given as median with interquartile ranges [square brackets]. FG, fluoroscopy-guided; Ro, robot-assisted; TLIF, transforaminal lumbar interbody fusion; AP, anteroposterior. * *p* < 0.05.

**Table 4:** Parameters of pedicle screws in screw loosening and non-screw loosening group.

	Screw Loosening Group (*n* = 48)	Non-Screw Loosening Group (*n* = 504)	*p* Value
Distance from pedicle screw to upper endplate/ vertebral body height	0.40 [0.38, 0.41]	0.37 [0.34, 0.41]	<0.001 *
Distance from screw tip to anterior cortex/ vertebral body AP diameter	0.16 [0.06, 0.25]	0.13 [0.05, 0.20]	0.100

Values are given as median with interquartile ranges [square brackets]. AP, anteroposterior. * *p* < 0.05.

**Table 5:** Screw loosening rate risk factors were established using the logistic regression model.

Components of Regression Model	Odds Ratio per Unit Change [95% CI]	*p* Value
Age	1.12 [1.04, 1.20]	0.001 *
Sex		
Female	Reference	
Male	1.19 [0.42, 3.35]	0.749
BMI	0.91 [0.80, 1.04]	0.177
Diagnosis		
Spinal stenosis	Reference	
Spondylolisthesis	0.93 [0.29, 2.93]	0.898
Spondylolysis	0.59 [0.06, 5.58]	0.647
Other degenerative disease	2.67 [0.40, 17.98]	0.314
Number of level(s) fused		
1	Reference	
2	2.52 [0.76, 8.37]	0.131
3	6.93 [1.45, 33.09]	0.015 *
Screw type		
Hollow screw	Reference	
Solid screw	1.17 [0.41, 3.35]	0.768
Cage type		
Polyetheretherketone	Reference	
Trabecular Metal	0.63 [0.17, 2.40]	0.502
Distance from pedicle screw to upper endplate/vertebral body height	63.24 [1.83, 2181.2]	0.022 *
Distance from screw tip to anterior cortex/vertebral body AP diameter	2.09 [0.23, 18.75]	0.509

BMI, body mass index; AP, anteroposterior. * *p* < 0.05.

**Table 6:** Patient-reported outcomes comparing delta VAS-Back, VAS-Leg, and ODI between FG TLIF and Ro TLIF group.

	FG TLIF Group (*n* = 79)	Ro TLIF Group (*n* = 29)	*p* Value
△VAS-Back-1	−4.0 [−6.0, −3.0]	−5.0 [−7.0, −3.0]	0.321
△VAS-Back-3	−5.0 [−7.0, −3.0]	−6.0 [−7.0, −4.0]	0.569
△VAS-Back-6	−5.0 [−7.0, −3.0]	−6.0 [−8.0, −4.0]	0.392
△VAS-Back-12	−5.0 [−7.0, −3.0]	−6.0 [−8.0, −3.0]	0.304
△VAS-Leg-1	−5.0 [−6.0, −3.0]	−6.0 [−8.0, −4.0]	0.137
△VAS-Leg-3	−5.0 [−7.0, −3.0]	−6.0 [−8.0, −4.0]	0.237
△VAS-Leg-6	−6.0 [−8.0, −4.0]	−7.0 [−8.5, −4.0]	0.278
△VAS-Leg-12	−6.0 [−8.0, −4.0]	−7.0 [−8.0, −3.0]	0.936
△ODI-1	−11.11 [−17.78, −4.45]	−13.33 [−21.11, −2.22]	0.579
△ODI-3	−20.00 [−26.66, −11.11]	−20.00 [−30.00, −7.78]	0.768
△ODI-6	−24.44 [−35.55, −13.34]	−26.67 [−35.56, −16.66]	0.456
△ODI-12	−26.67 [−35.56, −15.56]	−31.11 [−38.89, −23.33]	0.295

△VAS-Back-1, difference between VAS-Back at postoperative 1 month and preoperation; △VAS-Back-3, difference between VAS-Back at postoperative 3 months and preoperation, etc. FG, fluoroscopy-guided; Ro, robot-assisted; TLIF, transforaminal lumbar interbody fusion; VAS, visual analog scale; ODI, Oswestry Disability Index.

## Data Availability

All data are available upon reasonable request from the corresponding author.
